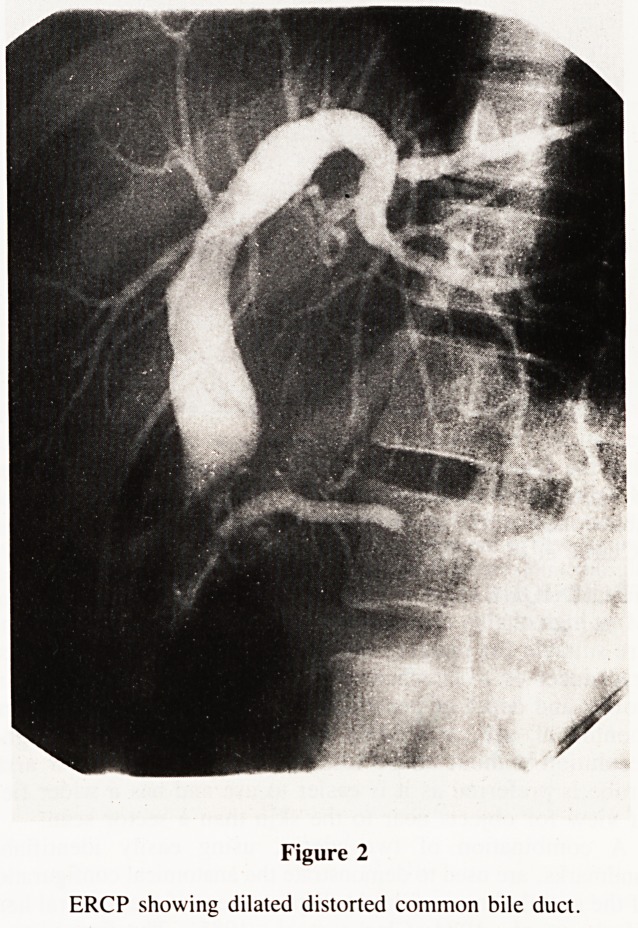# Malignant Neuro-Endocrine Tumour of the Pancreas, Salmonella Enteritidis Cholangitis and Pseudomembranous Cholecystitis

**Published:** 1991-09

**Authors:** Andrew Skyrme-Jones, Stephen Hughes, Patricia A. Burton, Graham V. N. Appleton, David J. Leaper

**Affiliations:** House Surgeon Department of Surgery, Southmead Hospital, Bristol BS10 5NB; Consultant Physician Department of Medicine, Southmead Hospital, Bristol BS10 5NB; Consultant Histopathologist Department of Pathology, Southmead Hospital, Bristol BS10 5NB; Lecturer Department of Surgery, Southmead Hospital, Bristol BS10 5NB; Consultant Surgeon Departments of Surgery, Southmead Hospital, Bristol BS10 5NB


### Malignant Neuroendocrine Tumour of Pancreas, Salmonella Enteritidis Cholangitis and Pseudomembranous Cholecystitis

**Andrew Skyrme-Jones, MB+, ChB** House Surgeon
**Stephen Hughes, MD*, MRCP** Consultant Physician
**Patricia A. Burton, MB, ChB, FRCPath****
Consultant Histopathologist
**Graham V. N. Appleton, MA, ChM, FRCS+** Lecturer
**David J. Leaper, MD+,ChM, FRCS** Consultant Surgeon

Departments of Surgery+, Medicine* and Pathology**
Southmead Hospital, Bristol BS10 5NB

Correspondence: Mr. D. J. Leaper, University Department of Surgery, The Medical School Unit, Southmead Hospital, Westbury-on-Trym, Bristol BS10 5NB.

This report presents a patient with several unique associations. Her underlying disease was a malignant neuroendocrine tumour of the pancreas but she presented with Salmonella enteritidis cholangitis and septicaemia and necrotising pseudomembranous cholecystitis.

### CASE REPORT
A 20 year old secretary was admitted with a three week history of diarrhoea, vomiting, steatorrhea, dark urine, abdominal pain and rigors. She was jaundiced, her pulse rate was 140/min and she had a temperature of 40°C. She had right upper quadrant peritonism with a positive Leake's sign.[1] She had a white cell count of 13.3 x $10^{12}$/l, a haemoglobin 10.3 g/dl, platelets 446 x $10^9$/l, prothrombin time 36s, thrombin time 26s, albumin 30 g/l, bilirubin 109 mmol/l, alkaline phosphatase 626 iu/l and a normal serum amylase. Ultrasound scan showed a 1 cm diameter common bile duct (CBD), a normal gallbladder with no stones. Hepatitis B virology was negative.

After six hours of resuscitation and treatment with IV cefuroxime and metronidazole, Vitamin K and fresh frozen plasma (FFP) she developed increasing tachypnoea. A chest X-ray (normal on admission) showed bilateral alveolar oedema. Her $paO_2$ was 4.6 kpa and $paCO_2$ 5.0 kpa, consistent with adult respiratory distress syndrome. At 12 hours, disseminated intravascular coagulopathy became evident (fibrinogen degradation products 2mg/l, fibrinogen 146 mg/dl, platelets 165 x $10^9$/l). Further FFP was given with 6 units of cryoprecipitate.

At 18 hours she required dopamine and dobutamine for refractory shock and oliguria. Laparotomy was carried out because of the signs of peritonitis. An inflamed, necrotic gallbladder (without stones) was found. The head of the pancreas was enlarged and rubbery but there was no pancreatitis. Cholangiography confirmed a wide CBD with a complete obstruction to contrast adjacent to the duodenal ampulla. A Harris catheter passed easily into the duodenum from a supraduodenal choledochotomy and choledochoscopy showed no ductal abnormality. Following cholecystectomy, the CBD was drained with a T-tube.

Histology of the gallbladder showed an oedematous wall with haemorrhage, inflammatory cell infiltrate and a mucosal pseudomembrane. Salmonella enteritidis (phage type 4) was isolated from the blood, faeces and operative bile samples. Antibiotics were changed to ciprofloxacin. She made a good recovery over 4 weeks although T-tube cholangiography showed the CBD to be blocked by lobulated indentations in its medial wall (Fig.1). CT scan revealed a mass in the head of the pancreas and ERCP revealed an ampullary tumour with a dilated, distorted CBD (Fig. 2). Biopsy of the ampulla showed malignant cells. Coeliac and superior mesenteric artery angiograms were normal.

A Whipple's procedure was carried out four weeks after the initial surgery. A tumour in the pancreatic head was confirmed. There was no evidence of extra-pancreatic disease or metastatic spread. Histology showed a malignant neuroendocrine tumour of the pancreas which stained with neurone specific enolase (NSE) and human chorionic gonadotrophin (HCG). The resection lines were clear.

The patient was discharged two weeks postoperatively. In view of the possibility of multiple endocrine neoplasia (MEN) type I postoperative investigations included skull X-ray, serum thyroxine, calcium, parathormone and prolactin, and urinary calcium. These were normal. Family history was unobtainable as the patient had been adopted.

### DISCUSSION

Our case illustrates several interesting and unusual associations. Salmonella have been isolated in Caroli's syndrome[2, 3] congenital hepatic fibrosis[4, 5], and cholangiocarcinoma[6], but cholangitis due to Salmonella infection is rare. As a corollary, recurrent cholangitits may occur in a typhoid carrier.[7] Salmonella enteritidis (phage type 4) caused an epidemic of gastroenteritis due to egg consumption[8], and was associated with the recent poultry scare.[9] No source of Salmonella was found in our patient or her family.

A pseudomembrane together with acute cholecystitis has been reported previously[10,11] but these patients had cholelithiasis and organisms were not isolated from bile.

Pancreatic endocrine tumours derived from APUD cells usually present with metabolic syndromes related to secreted hormones rather than by effects of local compression (typical of adenocarcinomas). The association of an endocrine tumour of the pancreas with MEN type I occurs with anterior pituitary tumours (in 65%) and parathyroid tumours (in 90%). This patient's tumour had NSE and HCG staining cells but there was no clinical evidence of hormone hypersecretion.

We should like to thank Professor J. Polak for her histochemical assistance.

### REFERENCES

1. JOHNSON A.G. The Gallbladder and Bile Ducts. In: Bailey and Love's Short Practice of Surgery. 15th edition (Eds. Rains A. J., Capper W. M.). London H. K. Lewis 1971 p852.
2. WALDRAM R.M.R., VAHRMAN J, WILLIAMS R. Salmonella heidelberg infection in Caroli's syndrome. *Gastroenterology* 1975; **68:** 151-153.
3. HAMLYN A.N., JAMES O.F.W., DOUGLAS A.P., LAVELLE M.I., VENABLES C.W. Caroli's disease with intrahepatic gallstones and Salmonella infection. *Postgraduate Medical Journal* 1976; **52:** 659-662.
4. SANCHEZ C, GONZALEZ E, GARAU J. Trimethoprim-sulfamethoxalzole treatment of cholangitis complicating congenital hepatic fibrosis. *Pediatric Infectious Diseases* 1986; **5:** 360-363.
5. UNITE I., MAITEM A., BAGNASCO F.M., IRWIN G.A.L. Congenital hepatic fibrosis associated with renal tubular ectasia. *Radiology* 1973; **109:**565-570.
6. ROBBINS S., CHUANG U.P. HERSH T. The development of hepatobiliary cancer in a carrier of Salmonella typhi. *Am J Gastroenterology* 1988; **83:** 675-678.
7. McFADZEAN A.J.S., ONG G.B. Intrahepatic typhoid carriers. *British Medical Journal* 1966; **1:** 1571-1574.
8. Editorial Salmonella enteritidis phage type 4: Chicken and Egg. *Lancet* 1988; **ii:** 720-722.
9. COYLE E.F., PALMER S.R., RIBEIRO C.D., et al. Salmonella enteritidis phage type 4 infection: assocation with hen's eggs. *Lancet* 1988; **ii:** 1295-6.
10. WALES L.R. Desquamated gallbladder mucosa: Unusual sign of cholecystitis. *Am J Roentgenol* 1982; **139:** 810-811.
11. CHEONG L.L., KHAN A.N., ARMSTRONG G.R. Ultrasonic demonstration of an intraluminal pseudomembrane in the gallbladder: A definitive sign of acute cholecystitis? *J. Clin Ultrasound* 1989; **17:** 449-452.

**Figure 1**

T-tube cholangiogram showing obstructed common bile duct with indentations of the medial wall.

**Figure 2**

ERCP showing dilated distorted common bile duct.

## Figures and Tables

**Figure 1 f1:**
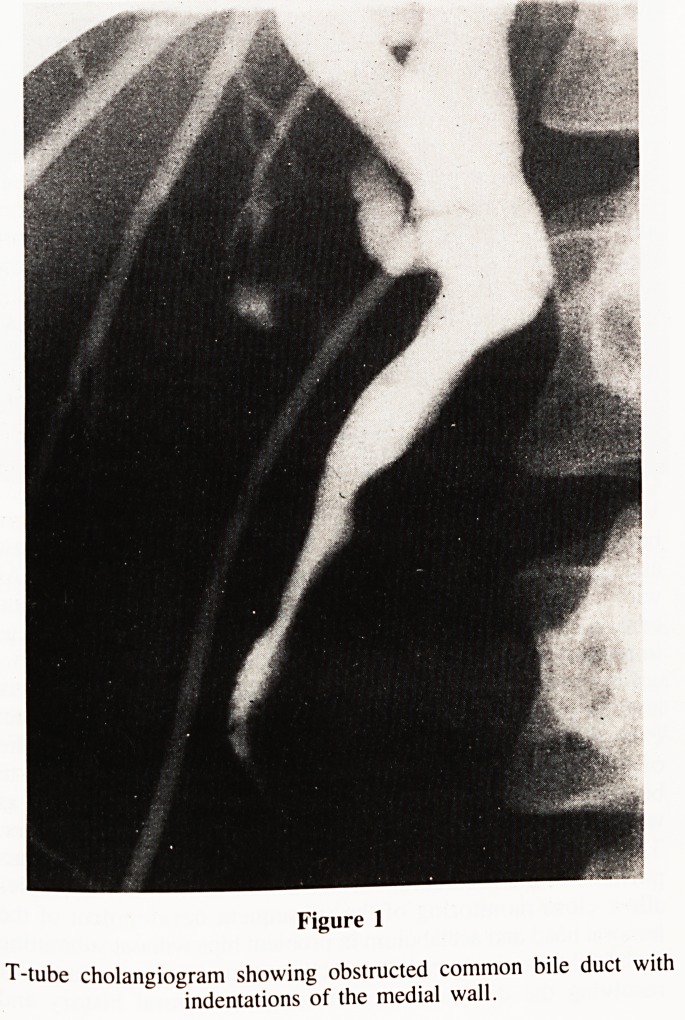


**Figure 2 f2:**